# Self-Calibration for the Time Difference of Arrival Positioning

**DOI:** 10.3390/s20072079

**Published:** 2020-04-07

**Authors:** Juri Sidorenko, Volker Schatz, Dimitri Bulatov, Norbert Scherer-Negenborn, Michael Arens, Urs Hugentobler

**Affiliations:** 1 Fraunhofer Institute of Optronics, System Technologies and Image Exploitation IOSB, 76275 Ettlingen, Germany; volker.schatz@iosb.fraunhofer.de (V.S.); dimitri.bulatov@iosb.fraunhofer.de (D.B.); norbert.scherer-negenborn@iosb.fraunhofer.de (N.S.-N.); michael.arens@iosb.fraunhofer.de (M.A.); 2 Institute of Astronomical and Physical Geodesy, Technical University of Munich, 80333 München, Germany; urs.hugentobler@tum.de

**Keywords:** dimension lifting, self-calibration, time-difference-of-arrival (TDOA)

## Abstract

The time-difference-of-arrival (TDOA) self-calibration is an important topic for many applications, such as indoor navigation. One of the most common methods is to perform nonlinear optimization. Unfortunately, optimization often gets stuck in a local minimum. Here, we propose a method of dimension lifting by adding an additional variable into the $l^{2}$ norm of the objective function. Next to the usual numerical optimization, a partially-analytical method is suggested, which overdetermines the system of equations proportionally to the number of measurements. The effect of dimension lifting on the TDOA self-calibration is verified by experiments with synthetic and real measurements. In both cases, self-calibration is performed for two very common and often combined localization systems, the DecaWave Ultra-Wideband (UWB) and the Abatec Local Position Measurement (LPM) system. The results show that our approach significantly reduces the risk of becoming trapped in a local minimum.

## 1. Introduction

Localization requires knowledge about the reference system, such as worldwide navigation satellites. The position of the satellites is well known, and it is unlikely that one satellite will disappear and reappear in a completely different orbit. This is different for most ground localization systems. If our own position is observed with respect to the reference system, it is necessary to know the coordinates of the reference system at any time. These coordinates can be obtained by external measurement equipment or by the system itself. The second case is also known as self-calibration. Self-calibration has the advantage that no additional measurement equipment is required, only the distance measurements between the reference stations and the changing tag position. Autonomous driving is a fascinating, albeit fiercely discussed, paradigm shift in today’s world of science. Contrary to other research fields of machine vision [1], it has already been established that the performance of automatic methods outperforms that of a human operator. However, while driving, humans are understandably reluctant to relinquish control. The crucial reason is that the problem of automatic self-localization in GPS-denied areas, such as urban canyons, has not yet been solved to a satisfying level, and much less so in dynamic environments where vehicles must consider not only static obstacles, but also each other. Signal-based self-localization has increasingly gained popularity. Two very common and often combined systems are the DecaWave Ultra-Wideband (UWB) and the Abatec Local Position Measurement (LPM) system. The DecaWave UWB system is less affected by reflections, but is, due to regulations, limited in its transmitting power. The LPM system faces the opposite problem. However, here, the problem of self-localization becomes problematic, where neither the positions of the transmitters nor the receivers are known. This is analogous to the microphone-speaker problem, where systems of (sometimes redundant or self-contradicting) quadratic equations must be solved, sometimes resulting in zero and sometimes resulting in dozens of solutions for the minimum cases (see Chapter 10 in [2]). In the few related works outlined below, it is therefore commonplace to find overdetermined systems of equations. From a reliable solution and a suitable optimizer, these systems are developed to converge to the global minimum and thus successfully self-localize the sensors.

The notations used in the text and in the equations are shown in Table 1 and Table 2.

## 2. Related Work

The most common approach for self-calibration is to perform nonlinear optimization [3]. This solution has the disadvantage that if the initial estimates are unfavorable, the optimization becomes stuck in a local minimum [4]. Different approximation strategies have been developed to reduce this risk. Wendeberg et al. [5,6] used an iterative cone alignment algorithm for the iterative solving of a nonlinear TDOA optimization problem through a physical spring-mass simulation. In [7], this problem was re-formulated by a linear rank constraint on the matrix, and the unknown variables were estimated by minimizing the cost function using nuclear norm minimization. A non-iterative algorithm with rank constraints was presented in [8,9]. In [10,11], Semidefinite Programming (SDP) was proposed as an initialization for the Maximum Likelihood (ML) estimator. As an alternative to iterative approaches, closed-form solutions have also been developed. Closed-form solutions were presented in [12], which used time-of-arrival information recovery and time-of-arrival-based joint source and sensor localization. Time-of-arrival self-calibration closed-form solutions were the subject of the investigation in [13,14,15].

However, approximations of the nonlinear model have different disadvantages. For example, Semidefinite Programming (SDP) [10,11] requires high computational power, and linear solutions are only valid for small measurement noise due to the need to square the nonlinear measurement equations [9]. Moreover, it is possible that sensor specifications will lead to nonlinear constraints, which will make it difficult to obtain a linear approximation. In the field of TDOA self-calibration, there are many other different methods, each dealing with a different aspect, such as the occurrence of an additional time offset [16]. None of these approaches, however, employ lifting of the objective function, which, as we show later, can always be applied to time-of-flight measurements.

### 2.1. Localization Systems

**Figure 1:**
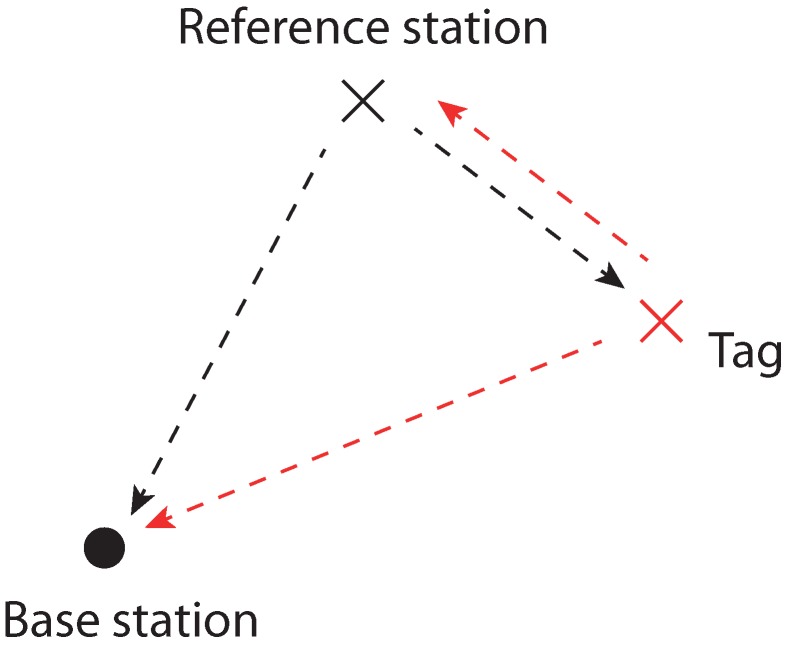
Transmitted messages with the DecaWave UWB system are represented by the dashed lines. The black circle is the base station, the black cross the reference station, and the red cross the tag.

**Figure 2:**
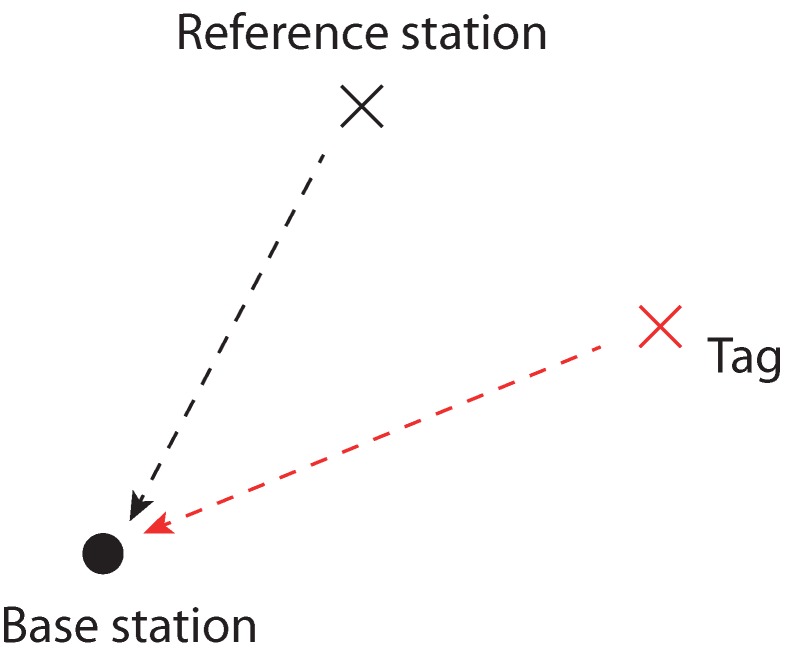
The messages transmitted with the Abatec Local Position Measurement (LPM) system are represented by the dashed lines. The black circle is the base station, the black cross the reference station, and the red cross the tag.

Dimension lifting has been tested with two different localization systems, the DecaWave Ultra-Wideband (UWB) and the Abatec Local Position Measurement (LPM) system. Both systems have a slightly different objective function. Figure 1 and Figure 2 illustrate the communication between the stations. The reference station and the tag emit a signal. The Base Stations (BS) obtain the difference between the arrival times of the two signals, which is also the reason why this technique is called the Time-Difference-Of-Arrival (TDOA). In contrast to the LPM system, the UWB system allows using the information about the flight time between the reference station and the tag. Moreover, the Abatec LPM objective function has an unknown offset $O_{j}$ for every emitted transmitter signal.

### 2.2. Mathematical Formulation

The regular objective functions are expanded by $\tilde{\lambda_{i}}$ for the base stations and tag $\lambda_{j}$ for the tags in the $l_{2}$ norm. This means that if the model is two-dimensional (i.e., the tag and the base stations are located on a two-dimensional plane), the objective function is expanded by a third dimension. This approach can be illustrated geometrically by imagining two circles. Both intersection points are the minima: one of them is the local minimum, and the other is the global optimum. If the optimization algorithm starts close to the local minimum and remains in the two-dimensional plane, it is not able to find an alternative path. With the additional dimension, the optimization algorithm is able to move along the intersection line of the spheres from the local minimum to the global optimum. The self-calibration will only use the change of the tag position to estimate the unchanging position of the reference station and base stations. Every position change of the tag increases the number of equations, as well as the number of the unknown $x_{j},y_{j}$, and $z_{j}$ coordinates of the tag and the time offsets $O_{i}$ for the LPM system. Since the positions of the base stations are unknown, the constellation can only be determined up to rotation and translation. It is necessary to keep in mind that the initial estimates should not be the same as or equal to zero.

The following two paragraphs show the coordinates of the transponder and the base stations for the classic approach and our lifted approach. Here, the only differences are the additional dimensions $\tilde{\lambda_{i}}$ and $\lambda_{j}$.

Classic approach:
$t_{j}=\left(\begin{array}{c} x_{j}\\ y_{j}\\ z_{j} \end{array}\right)$
$b_{i}=\left(\begin{array}{c} a_{i}\\ b_{i}\\ c_{i} \end{array}\right)$

Our lifted approach:
$t_{j}=\left(\begin{array}{c} x_{j}\\ y_{j}\\ z_{j}\\ \lambda_{j} \end{array}\right)$
$b_{i}=\left(\begin{array}{c} a_{i}\\ b_{i}\\ c_{i}\\ {\tilde{\lambda }}_{i} \end{array}\right)$

## 3. TDOA Localization

In [17], we demonstrated that if the base station positions are known and only the tag positions have to be estimated, an additional dimension in the $l^{2}$ norm of the TOA objective function transforms the local minimum to a saddle point. This fact has been proven analytically for the squared objective function and empirically for the general TOA objective function through more than 10,000 test scenarios with different constellations and initial estimates. The test scenarios were repeated for the general TDOA equation. Table 3 shows the results of 10,000 nonlinear optimizations with the presented TDOA measurements. In contrast to the self-calibration, the results in Table 3 are provided with known base station positions. The optimization was performed with four base stations for the UWB objective function and with five base stations for the LPM objective function. The lifted UWB objective function did not converge at a single time to the local minimum instead of the correct global minimum. On the other hand, this did not apply for the LPM equation. The difference was that the offset also needed to be estimated for the LPM equation. This led to the lifted LPM optimization being inferior to the lifted UWB optimization. The same applied if one LPM measurement was subtracted from another LPM measurement with the aim of eliminating the offset. The results of the subtracted optimization are presented in the final column of Table 3.

## 4. TDOA Self-Calibration

In the previous section, the positions of the base and reference station were known. In this section and the following section, the base and reference station positions are unknown. The self-calibration presented for the UWB and LPM system was based on measurements between the base stations, the reference station, and the tag. Since the base stations were passive, the distance measurements between the base stations were unknown. An important factor to determine for self-calibration is how many measurements should be used for optimization. Usually, the answer is as many as possible, as using more measurements reduces noise. In contrast to the fully-numerical method, the number of unknown variables does not increase with more measurements for the partially-analytical method. Table 4 shows the ratio between the number of equations and the unknown variables. The ratio of the partially-analytical method increased indefinitely with the number of measurements. In contrast to the LPM objective function, the UWB objective function was the reference station portion of the base stations. In the presence of noise and nonlinear constraints, nonlinear optimization was the solution of choice. The results were determined with the MATLAB Levenberg–Marquardt algorithm.

### 4.1. Objective Functions

The DecaWave UWB minimization of the objective function is:

(1) $$\mathrm{argmin}\left(\sum_{i=1}^{N}\sum_{j=1}^{M}{\left[∥T_{j}-B_{i}∥-∥B_{1}-B_{i}∥-d_{i,j}\right]}^{2}\right)$$

Abatec LPM minimization of the objective function is:

(2) $$\mathrm{argmin}\left(\sum_{i=2}^{N}\sum_{j=1}^{M}{\left[∥T_{j}-B_{i}∥-∥B_{1}-B_{i}∥+O_{j}-d_{i,j}\right]}^{2}\right)$$

### 4.2. Fully-Numerical Method

In the first method, the objective functions from Equations (1) and (2) were used. The ratio (Table 4) approached $\frac{N}{D}$ for UWB and $\frac{N}{D+1}$ for LPM with M→∞.

### 4.3. Partially-Analytical Method

In this method, only the base station positions were obtained by non-linear optimization. These positions were used to obtain the tag positions analytically in every iteration step. The ratio Ra linearly increased with *M*. In contrast to the UWB, it was necessary to calculate the offset analytically for the LPM, as well. The linear estimation that we used can be found in [18]. This linear solution was expanded by the ability to operate with the additional dimensions $\tilde{\lambda_{i}},\lambda_{j}$ and $\bar{\lambda }$. Like with the fully-numerical method, it was necessary to keep in mind that the initial estimates should not be the same as or equal to zero.

## 5. TDOA with the Decawave UWB System

In the following, the two test methods are employed to UWB self-calibration with and without the additional dimension. The coordinate system was centered on the position of the reference station $a_{ref}=b_{ref}=c_{ref}={\tilde{\lambda }}_{ref}=0$.

### 5.1. Synthetic Data

#### 5.1.1. Random Geometry

Table 5 shows the number of false optimizations with synthetic data. The evaluation of the optimization was undertaken using the mean squared error between all objects provided by the optimization and the ground truth distances. The base stations $B_{i}$, tag $T_{j}$, and the initial estimates were randomly generated in a 10×10×10 cube. For every test case, ten-thousand constellations were created and tested with the Levenberg–Marquardt algorithm.

The number of false results increased with more measurements using the fully-numerical method, whereas it decreased or remained the same for the partially-analytical method. This did not apply for the lifted objective functions. The false rate decreased or remained the same for the partially-analytical method. This did not apply to the lifted objective functions. With more measurements, the false rate decreased with the lifted fully-numerical method in contrast to the lifted partially-analytical method. With a higher number of base stations, the false result rate between the two methods became the opposite. Put simply, with a higher number of base stations, it was recommended to use the lifted fully-numerical method or the lifted partially-analytical method.

#### 5.1.2. Selected Geometry

The geometric constellation, which is known as the Dilution Of Precision (DOP), and the noise/outliers had a strong impact on localization. Therefore, the test was repeated using synthetic data without noise and outliers, but with the same geometric constellation as the real measurements, as shown in Table 6. Equivalent to the real measurement data was the position of the tag, which changed 23 times. The optimization was repeated 10,000 times, with random initial estimates.

### 5.2. Real Measurements

**Figure 3:**
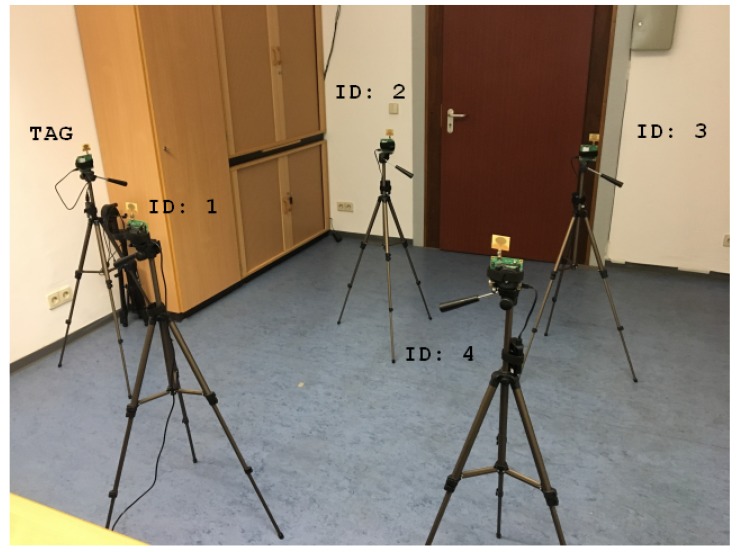
Experimental setup with the DecaWave UWB. The four UWB base stations with ID 1 to 4 are mounted on a tripod. The tag is located on the left side in the picture.

**Figure 4:**
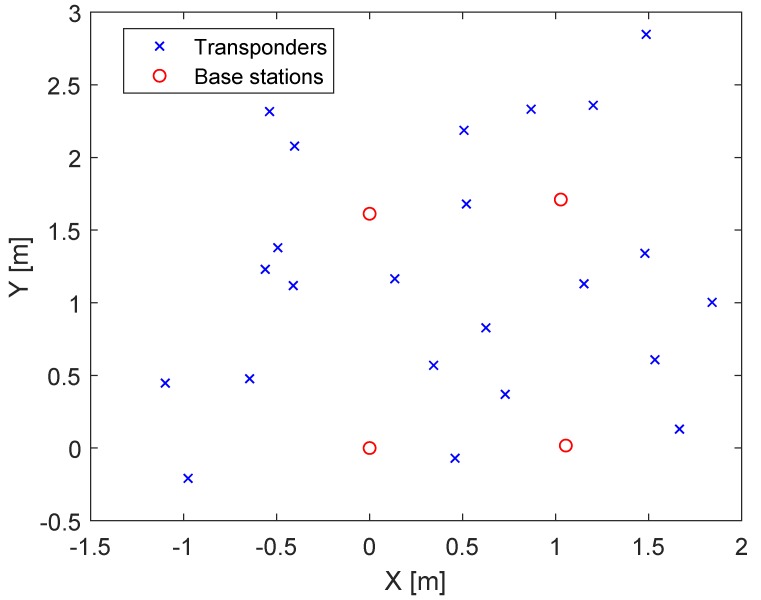
Constellation of the DecaWave UWB systems. The red circles are the real base stations locations, and the crosses are the tag locations.

In this section, the previously obtained synthetic results for the UWB objective function are verified by the measurements received from the sensor data. The DecaWave transceivers were based on Ultra-Wideband (UWB) technology and complied with the IEEE802.15.4-2011 standard [19]. They supported six frequency bands, with center frequencies ranging from 3.5 GHz to 6.5 GHz and data rates of up to 6.8 Mb/s. The bandwidth varied with the selected center frequencies, from 500 to 1000 MHz. The timestamps for positioning were provided by estimating the Channel Impulse Response (CIR). The CIR estimation was obtained by correlating a known preamble sequence against the received signal and accumulating the results over a certain period of time. In contrast to narrow band signals, the UWB was more resistant to multipath fading [20]. Reflections caused an additional peak in the impulse response. The probability that two peaks interfered with each other was small. The sampling of the impulse response was performed by an internal 64 GHz chip with 15 ps event timing precision (4.496mm). Due to general regulations, the transmission power density was limited to −41.3 dBm/MHz. These regulations were due to high bandwidth occupied by the UWB transceiver. The following experiments were performed using a DecaWave EVK1000. This board mainly was comprised of a DW1000 chip and a STM32 ARM processor. Equation (1) shows the objective function of our UWB TDOA equation. The base station with ID 1 was used as the reference station with the coordinates $a_{1},b_{1}$ and $c_{1}$. In contrast to the LPM, the UWB objective function contained one additional measurement due to the distance measurement between the tag and the reference station. Table 6 and Figure 3 show the constellation of the base stations, the reference station, and the tag. The ground truth distances were obtained by a laser rangefinder. The station with ID 1 was the reference station. The position of the tag was changed 23 times. Every distance measurement was based on the mean of 2000 measurements in one position. The optimization was repeated 10,000 times with random initial estimates. Figure 4 shows a constellation of the base stations and the tag positions. The results of the optimization with real measurements can be found in Table 7. In contrast to the synthetic results, the lifted partially-analytical method performed less favorably than the non-lifted method. The best results were delivered by the lifted fully-numerical method with 23 tag measurements. This discrepancy with the synthetic measurements was due to the noise and the outliers. In Table 8, the test was repeated with synthetic data, but with the same geometric constellation of the base stations and transponders, like with the real case. The only differences between the two test cases were the noise and the outliers. It can be observed that the constellation from Table 6 required fully-numerical optimization. The performance of the partially-analytical method primarily depended on the intermediate solution provided by the linear estimator. Outliers had a much stronger influence on the linear solution than on nonlinear optimization. More information about the effects of noise on optimization with an additional dimension was provided in [21].

## 6. TDOA with the Abatec LPM System

In this section, LPM self-calibration is obtained for the fully-numerical and partially-analytical method, with and without the additional dimension. The coordinate system is centered at the reference station.

### 6.1. Synthetic Data

The test scenarios for the synthetic data were equivalent to those for UWB optimization. The positions of the base stations $B_{i}$, tag $T_{j}$, and initial estimates were randomly generated in a 10×10×10 cube. For each *N* and *M*, ten-thousand constellations were created. Table 9 shows the results of the optimization, in which the offset was eliminated by subtracting one base station from all the other base stations. The offset could also be eliminated by subtracting all the base stations from each other. This made the objective function more symmetrical. The results of this optimization can be found in Table 10. Generally, the optimization in which the offset was eliminated by subtracting all stations from each other had slightly better results. In contrast to the UWB optimization, the number of false results increased for the lifted fully-numerical method with an increasing number of measurements. However, this did not apply to the lifted partially-analytical method. Optimization with the offset was not recommended since it was not possible to pre-filter the data. Furthermore, the optimization required much more time due to the additional unknown variables.

### 6.2. Real Measurements

**Figure 5:**
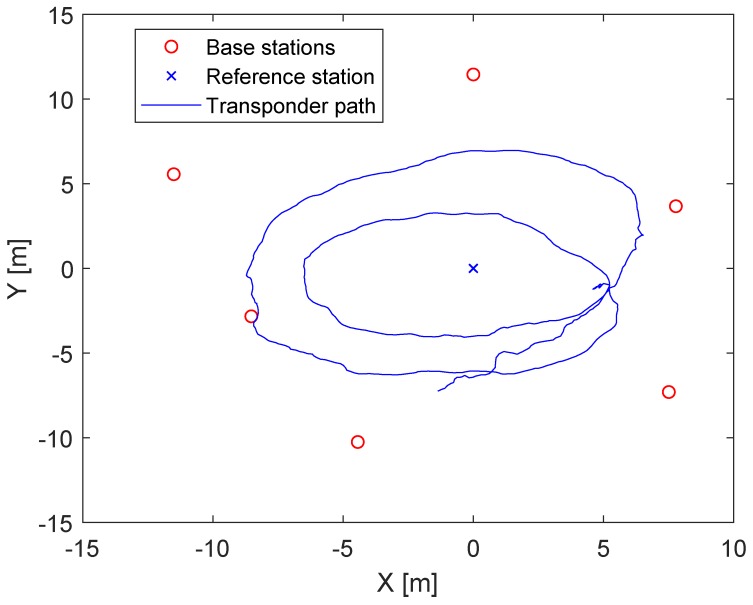
Constellation of the LPM system. Red circles are the base stations, the blue cross the reference station, and the blue line the tag path.

**Figure 6:**
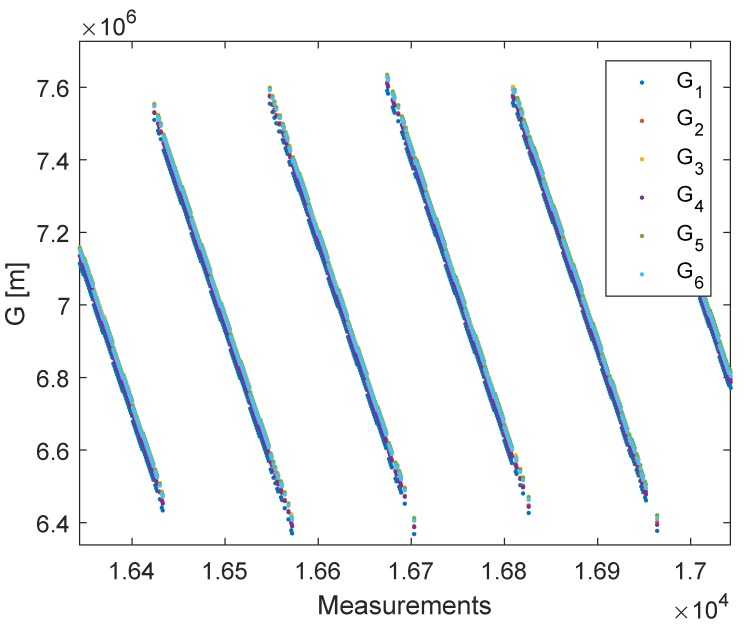
Zoom-in of the raw LPM measurements. The different colors represent the outcomes of the raw measurements before every station is subtracted from Station 1.

The real LPM measurements are the subject of the investigation in this section. Figure 5 shows the constellation of the base stations, the reference station, and the tag path. In contrast to the UWB measurement, the LPM measurements were now obtained while moving the tag. On the one hand, this approach was more practical because it allowed obtaining more measurements more quickly. On the other hand, more intelligent filters required were now needed to reduce noise and eliminate outliers. Additionally, it had to be taken into account that the tag was carried by hand; thus, it was not possible to provide a perfect two-dimensional plane. The Local Position Measurement system (LPM) by Abatec was strongly inspired by the Frequency-Modulated Continuous Wave radar (FMCW). This radar system generates an increasing frequency chirp, which is sent in a certain direction. In the next step, the reflected signal was compared with the internal chirp. The frequency difference between these chirps represented the range with respect to the slope of the chirp. Generally, the frequency differences were obtained by additive or multiplicative mixers. The LPM used the same principle; however, in contrast to the FMCW radar, the sent chirp of the tag was mixed with the internal chirp of the base station. The chirp itself had a bandwidth of 150 MHz and a ramp duration of 500 us, with an update rate of 1000 measurements per second [22]. If the starting times of the tag and the base station were synchronous, the result would be equivalent to that of the FMCW radar, although the range would be 50% shorter. The frequency difference represented the flight time, with the assumption that electromagnetic waves propagated with the speed of light and that we could obtain the relative range. Unfortunately, the base stations did not have the same starting times; hence, they were not synchronous, which led to a time offset. The Abatec LPM system used a reference station in a known fixed position. The position of the tag could be estimated for every measurement if we had four base stations and one reference station in known positions. Knowledge about the base station and reference station positions could be obtained from several measurements by changing the position of the tag. Accordingly, there was no need for further hardware to calibrate the system. The Abatec LPM system was described in detail in [22,23]. Previous publications on the Abatec LPM have been mainly concerned with the measurement principles [22,23] and how the sensor data can be fused and filtered to detect an outlier [24] and obtain the most accurate position [25]. The most recent publications on LPM have focused on the numerical solver. Generally, the LPM uses a Bancroft algorithm [26,27,28] to estimate the position of the tag. At this time, no work has been conducted on LPM self-calibration. In contrast to the DecaWave UWB, the LPM objective function (2) had an additional offset $O_{j}$, which was higher than the distance measurement by a factor of 1000. Figure 6 shows the raw LPM measurements, $G_{i}$, Equation (3).

(3) $$\begin{array}{c} G_{i}=\left(∥T_{j}-B_{i}∥-∥B_{r}-B_{i}∥+O_{j}\right) \end{array}$$

The only changing value was $T_{j}$, due to the moving tag, with the index *i* as the ID of the base station, index *r* as the used reference station (r≠i), and *j* as the tag position.

(4) $$\begin{array}{c} G_{1,i}^{S}=\left(∥T_{j}-B_{1}∥-∥B_{r}-B_{1}∥\right)\\ -\left(∥T_{j}-B_{i}∥-∥B_{r}-B_{i}∥\right) \end{array}$$

**Figure 7:**
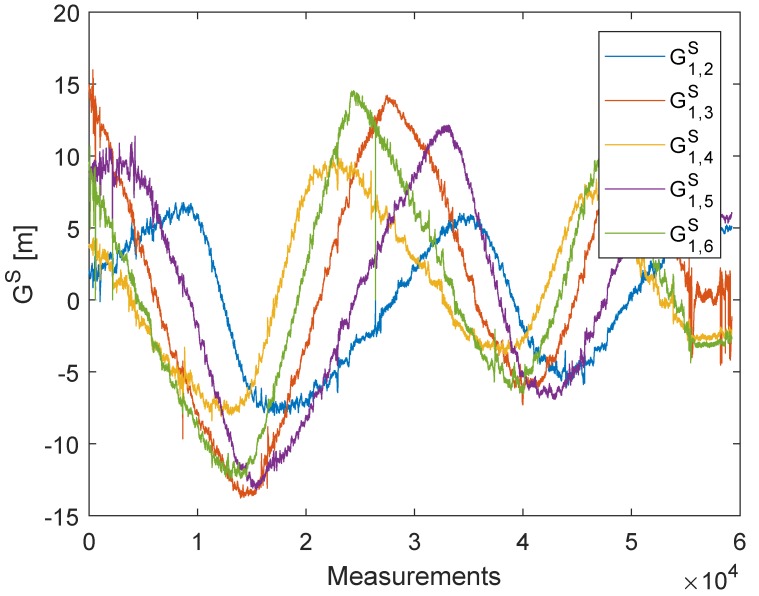
LPM measurement differences. The different colors represent the outcome of the filtered measurements, after every station is subtracted from Station 1.

The plot of Figure 7 shows the measurements, $G_{1,i}^{S}$, Equation (4), after subtracting one station from another to eliminate the offset.

The LPM system was more suitable for long-range measurements, although it was more strongly affected by reflections and fading [29]. This made it difficult to differentiate between the moving path and the measurement errors. After filtering, the measurement data were split into different subsets. The number of subsets equated to the number of tag positions *T*. In every optimization test, one measurement of the filtered data from every subset was randomly selected for optimization. Table 11 shows the results of 10,000 tests. The number of false results was much higher than that of the UWB TDOA self-calibration. The lifted partially-analytical method was thus not usable. The best results were provided by the fully-numerical method with six base stations and only fifteen measurements. With an increasing number of measurements, the possibility of false results also increased. In contrast to the synthetic data, the results of the lifted fully-numerical method were better than those of the general method, with a higher number of tags in some constellations. More measurements also had the advantage of reducing Gaussian noise. This would be the ideal method; however, in reality, there are always outliers. In practice, we recommend using the RANSAC algorithm [30]. The important disadvantage of the LPM equation was that all data were strongly affected by the offset $O_{j}$, which changed from one measurement to the next. In [18], we showed that subtracting one measurement from another eliminated the offset and had some significant advantages for data filtering. In contrast to the UWB, it was also necessary to calculate the offset analytically for the LPM. The linear estimation that we used can be found in [18]. This linear solution was expanded by the ability to operate with the additional dimensions, $\tilde{\lambda_{i}}$ and $\lambda_{j}$.

## 7. Conclusions

This paper presented a dimension-lifting approach to reduce the risk of converging to a local minimum during nonlinear optimization. The impact of the additional dimension in the $l^{2}$ norm on self-calibration was shown by synthetic and real measurements. Optimization was carried out using two different methods. In the fully-numerical method, all of the unknown parameters were optimized. In the partially-analytical method, the optimization handled only the base station position estimation, while the other unknown parameters were obtained analytically in each iteration step. The methods were evaluated by synthetic data and real measurements for the DecaWave UWB and Abatec LPM systems. The lifted TDOA method provided the best results for optimization with Gaussian noise and a sufficient quantity of base stations to compensate for the additional dimension. The additional dimension increased the number of unknown variables; therefore, it was necessary to obtain more measurements for an exactly determined or an overdetermined system. This did not apply to measurements with outliers; hence, it was required to pre-filter the real measurements before self-calibration.

## Figures and Tables

**Table 1:** Notations used in the text and in the equations.

Notations	Definition
$B_{i}$	Base stations, 1≤i≤N
*D*	Number of dimensions
*N*	Number of base stations $B_{i}$
*M*	Number of independent measurements $T_{j}$
Ra	Number of equations/number of unknowns
$T_{j}$	Tags, 1≤j≤M

**Table 2:** Notations used in the equations.

Notations	Definition	Status
**b${}_{i}=$** ${\left(a_{i},b_{i},c_{i}\right)}^{T}$	Position of base station $B_{i}$	Unknown
**t${}_{j}=$** ${\left(x_{j},y_{j},z_{j}\right)}^{T}$	Position of transponder $T_{j}$	Unknown
$d_{i,j}$	Distance measurements between $B_{i}$ and $T_{j}$	Known
$O_{i}$	Local Position Measurement (LPM) time offset	Unknown
$\tilde{\lambda_{i}}$	Additional dimension of $B_{i}$	Unknown
$\lambda_{j}$	Additional dimension of $T_{j}$	Unknown
*r*	Distance between the base and reference station	Unknown

**Table 3:** The self-localization results are based on a 2D model with synthetic data and known reference stations positions. The identified false optimizations have an error larger than 0.1.

False Result Rate (%)	UWB	LPM with Offset	LPM (Subtracted)
General TDOA	13.19	6.99	9.88
Lifted TDOA	0	0.10	0.08

**Table 4:** Ratio between the number of equations and the number of unknown variables.

	UWB	LPM
Ra Fully-Numerical	$\frac{N\cdot M}{D\cdot \left(N+M\right)}$	$\frac{N\cdot M}{D\cdot \left(N+M+1\right)+M}$
Ra Partially-Analytical	$\frac{M}{D}$	$\frac{N\cdot M}{D\cdot \left(N+1\right)}$

**Table 5:** The UWB self-calibration results are based on a 2D model with synthetic data. The identified false optimizations have an error larger than 0.1.

	N=4, T=23	N=5, T=9	N=5, T=18	N=5, T=27
Ratio Ra: fully-numerical	1.14	1.07	1.30	1.41
Ratio Ra: partially-analytical	7.6	3	6	9
False results: fully-numerical (%)	70.91	51.09	57.61	61.44
False results: partially-analytical (%)	48.75	55.97	55.41	55.76
False results: lifted fully-numerical (%)	7.71	5.46	2.73	2.03
False results: lifted partially-analytical (%)	8.13	11.68	10.28	10.64

**Table 6:** Coordinates of the UWB stations.

Station ID	X (m)	Y (m)	Z (m)
1	0	0	0
2	0	1.613	0
3	1.028	1.710	0
4	1.055	0.017	0

**Table 7:** UWB self-calibration optimization results based on real 2D measurements. The identified false optimizations have an error larger than 0.1m.

	N=4, T=23
Ratio Ra: fully-numerical	1.14
Ratio Ra: partially-analytical	7.6
False results: fully-numerical (%)	51.28
False results: partially-analytical (%)	55.41
False results: lifted fully-numerical (%)	20.90
False results: lifted partially-analytical (%)	99.92

**Table 8:** UWB self-calibration optimization results with synthetic measurements based on the same geometric constellation as the real 2D measurements. The identified false optimizations have an error larger than 0.1m.

	N=4, T=23
Ratio Ra: fully-numerical	1.14
Ratio Ra: partially-analytical	7.6
False results: fully-numerical (%)	33.37
False results: partially-analytical (%)	54.47
False results: lifted fully-numerical (%)	6.91
False results: lifted partially-analytical (%)	0.45

**Table 9:** The self-calibration results are based on a 2D model of synthetic LPM measurements where the offset is eliminated by subtracting one measurement equation from all others. The identified false optimizations have an error larger than 0.1.

	N=5, T=30	N=5, T=45	N=6, T=15	N=6, T=30	N=6, T=45
Ratio Ra: fully-numerical	1.08	1.14	1.1	1.28	1.34
Ratio Ra: partially-analytical	8.3	12.5	12.86	8.57	12.86
False results: fully-numerical (%)	83.30	88.06	63.51	74.34	81.32
False results: partially-analytical (%)	88.70	89.82	74.35	77.61	79.59
False results: lifted fully-numerical (%)	57.43	60.89	32.06	33.22	39.43
False results: lifted partially-analytical (%)	85.62	86.63	43.28	38.10	38.72

**Table 10:** Self-calibration results are based on a 2D model of synthetic LPM measurements where the offset is eliminated by forming a pairwise difference of measurement equations. The identified false optimizations have an error larger than 0.1.

	N=5, T=30	N=5, T=45	N=6, T=15	N=6, T=30	N=6, T=45
Ratio Ra: fully-numerical	1.08	1.14	1.1	1.28	1.34
Ratio Ra: partially-analytical	8.3	12.5	12.86	8.57	12.86
False results: fully-numerical (%)	78.81	84.61	58.43	66.83	74.92
False results: partially-analytical (%)	84.57	86.08	68.85	71.80	74.95
False results: lifted fully-numerical (%)	55.84	60.31	31.99	33.14	40.55
False results: lifted partially-analytical (%)	82.78	83.30	37.78	31.01	30.11

**Table 11:** Self-calibration results of the real LPM measurements. The identified false optimizations have errors larger than 0.1m.

	N=6, T=15	N=6, T=30	N=6, T=45
Ratio Ra: fully-numerical	1.1	1.28	1.34
Ratio Ra: partially-analytical	4.29	8.57	12.86
False results: fully-numerical (%)	79.49	92.21	96.41
False results: partially-analytical (%)	93.17	96.47	98.73
False results: lifted fully-numerical (%)	89.30	87.12	91.13
False results: lifted partially-analytical (%)	99.81	99.98	100
